# Changes in fractional exhaled nitric oxide, forced expiratory volume in one second, and forced oscillation technique parameters over three years in adults with bronchial asthma managed under Yokohama Seibu Hospital’s coordinated care system

**DOI:** 10.1186/s12890-024-03040-7

**Published:** 2024-05-02

**Authors:** Takahiro Tsuburai, Satoshi Tanaka, Yuko Komase, Baku Oyama, Hiromi Muraoka, Yusuke Shinozaki, Kazuhiro Nishiyama, Junko Ueno Shibuya, Yoshihiro Nishi, Yu Numata, Naoya Hida, Masamichi Mineshita, Takeo Inoue

**Affiliations:** 1 Department of Respiratory Medicine, St. Marianna University Yokohama Seibu Hospital, Yasashi-chou 1197-1, Asahi, Yokohama, Kanagawa 241-0811 Japan; 2 Department of Respiratory Medicine, St. Marianna University School of Medicine, Kawasaki, Japan

**Keywords:** Asthma, Coordinated care system, FeNO, fForced oscillation technique, Spirometry

## Abstract

**Background:**

In western Yokohama, our hospital and primary care clinics manage adults with asthma via a coordinated care system. We investigated the changes in the fractional expired nitric oxide (FeNO), forced expiratory volume in 1 second (FEV_1_), and forced oscillation technique (FOT) parameters over 3 years in a cohort of patients in our collaborative system.

**Methods:**

From 288 adults with well controlled asthma managed under the Yokohama Seibu Hospital coordinated care system between January 2009 and May 2018, we selected 99 subjects to undergo spirometry, FeNO and FOT testing over 3 years and analyzed the changes in these parameters.

**Results:**

Of the 99 patients enrolled, 17 (17.2%) experienced at least one exacerbation (insufficiently controlled (IC)), whereas, 82 (82.8%) remained in well controlled during the 3-year study period. Of well-controlled patients, 54 patients (54.5%) met the criteria for clinical remission under treatment (CR); the remaining 28 patients did not meet the CR criteria (WC). There were no differences in FeNO, FEV_1_, or FOT parameters at baseline among the IC, WC, and CR groups. The levels of FEV_1_ decreased gradually, whereas the levels of FeNO decreased significantly over 3 years. The levels of percent predicted FEV_1_ (%FEV_1_) significantly increased. We also observed significant improvement in FOT parameters; reactance at 5 Hz (R_5_), resonant frequency (Fres), and integral of reactance up to the resonant frequency (AX). The CR group demonstrated significant relationships between the change in FeNO and the change in FEV_1_ and between the change in FEV_1_ and the change in FOT parameters. No significant correlations emerged in the IC or WC group.

**Conclusion:**

The decrease in FeNO and increase in %FEV_1_, we observed in all study participants suggest that the coordinated care system model benefits patients with asthma. Although it is difficult to predict at baseline which patients will experience an exacerbation, monitoring changes in FeNO and FEV_1_ is useful in managing patients with asthma. Furthermore, monitoring changes in R_5,_ Fres, and AX via forced oscillation technique testing is useful for detecting airflow limitation.

**Supplementary Information:**

The online version contains supplementary material available at 10.1186/s12890-024-03040-7.

## Introduction

Asthma is a disease “characterized by chronic eosinophilic airway inflammation, which clinically manifests as variable bronchoconstriction (wheezing, dyspnea, chest tightness, and cough)” [1] and exacerbations of sudden, reversible airway narrowing. Treating with inhaled corticosteroids and managing acute exacerbation are important for controlling chronic inflammation and preventing deterioration in lung function. Creating an environment where it is easy for patients with asthma to adhere to their preventive treatment is essential. Given the high prevalence of asthma, it is reasonable for primary care doctors to manage patients who have stable disease. Furthermore, according to another recent study, approximately 30% of people with stable asthma treated by primary care doctors achieved remission when reassessed in 12 months [2]. However, it is difficult for primary care doctors to manage exacerbations at night or on holidays, conduct specialized examinations, or adequately evaluate and adjust asthma treatments.

In a previous study [3], researchers compared fractional expired nitric oxide (FeNO), forced expiratory volume in 1 second (FEV_1_), and symptom-based strategies as indicators for adjusting treatment in patients with mild-to-moderate asthma. Whereas each study indicator improved, others remained unchanged. The results highlighted the limitation of using a single assessment method to guide appropriate asthma management.

In recent years, patients with asthma have been better controlled with drugs, and the number of extremely stable patients is increasing. Although there is no current cure for asthma, the concept of “clinical remission”, as applied to other autoimmune diseases, has been proposed [4, 5]. Clinical remission is defined as the absence of symptoms or exacerbations for at least 12 months, and a percent predicted FEV_1_ (%FEV_1_) > 80%. Complete remission is further defined as the normalization of the underlying pathology. It is thought that a group of patients with asthma in the study cited above [2] had achieved clinical remission. Furthermore, that there are likely stable patients managed by primary care physicians who meet the criteria for clinical remission.

The forced oscillation technique (FOT) has attracted attention as a management method for asthma, because it enables providers to measure respiratory system resistance during rest ventilation non-invasively [6]. In support of its usefulness, we showed previously that among the FOT indicators, Fres correlated with reversibility in stable asthma [7]. We now theorize that the FOT indices may be useful in determining the degree of stability, in patients with stable asthma.

More than ten years ago, our hospital established a cooperative system of coordinated care with our primary care clinics to manage patients with asthma. We previously reported on the program's progress [8], but we could not predict which patients with controlled asthma were at risk for exacerbations. Prior reports suggested that increases in FeNO and large decreases in FEV_1_ may be useful predictors of exacerbations [9]. Thus, maintaining markers of Since residual airway inflammation within the target range is a necessary condition for effective asthma control [1].

The above evidence combined with our hospital experience led us to hypothesize that a) FeNO, FEV_1_, and FOT indices could be used to predict future exacerbations in patients receiving stable asthma management, and b) regular treatment review by primary care physicians could prevent future exacerbations. Guided by these hypotheses, we investigated whether FeNO, FEV_1_, and FOT indices could predict exacerbations over the 3-year study period and prevent respiratory function deterioration in adults with asthma managed by primary care physicians.

## Methods

This study was conducted at the Department of Respiratory Medicine, St. Marianna University Yokohama Seibu Hospital from January 2009 to May 2018. We recruited 99 patients (75 women) to be constantly followed with FeNO, FEV_1_, and FOT for 3 years from among 288 patients (201 women) in the coordinated care system for asthma who entrusted their usual care to primary care physicians (Table 1) [8].

**Table 1:**
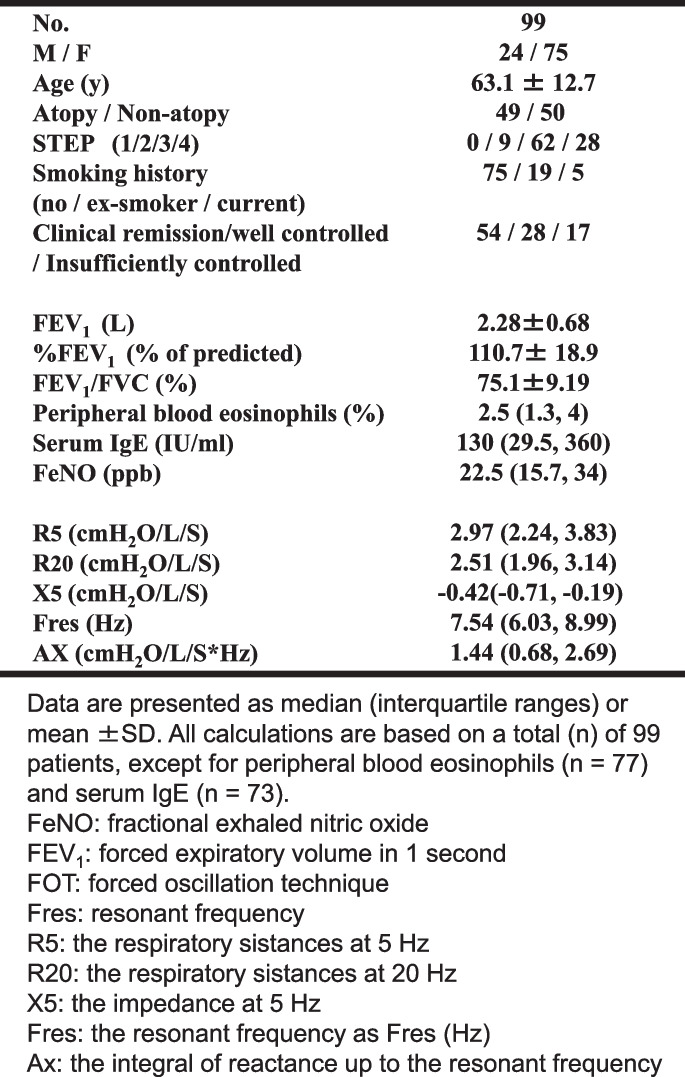
Baseline characteristics of patients of this study. FeNO: fractional exhaled nitric oxide. FEV_1_: forced expiratory volume in 1 second, FOT: forced oscillation technique, R_5_: the respiratory sistances at 5 Hz, R_20_: the respiratory resistances at 20 Hz, X_5_: the impedance at 5 Hz, Fres: the resonant frequency as Fres (Hz), Ax: the integral of reactance up to the resonant frequency

**Fig. 1:**
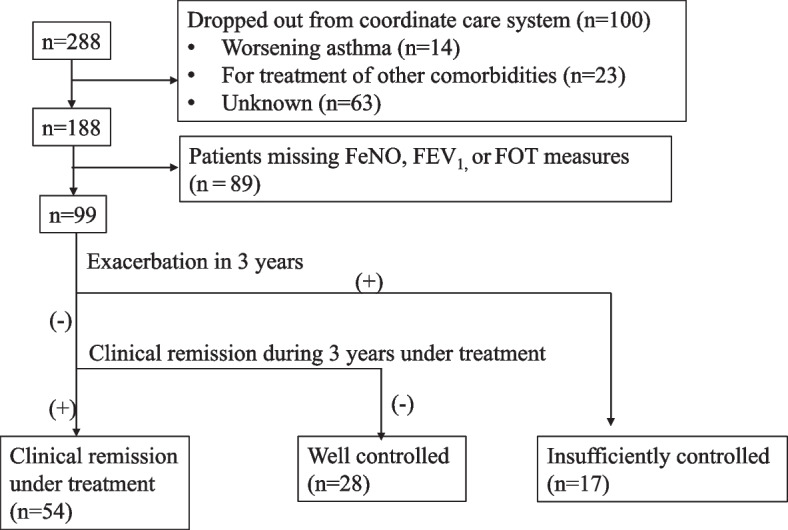
Patient characteristics at study enrollment

Asthma was diagnosed in patients with bronchodilator-responsive sudden cough, wheezing, and dyspnea according to the Asthma Prevention and Management Guideline in Japan at the time [1, 10, 11]. The physician in charge confirmed the diagnosis in patients with eosinophilic airway inflammation and reversible airway narrowing. Specialists in respiratory medicine prescribed medications based on the guideline. We excluded patients with respiratory disease complications, pregnant or lactating patients, and patients who were lost during follow-up. If one or more perennial inhalation allergens (house-dust, mite, dog, cat, *Aspergillus*, *Alternaria*, moth, cockroach, or midge) were positive, the patient was classified as atopic [1]. Usual medical care was provided by the primary care physician. An asthma exacerbation was defined as a) hospitalization, b) an unscheduled medical visit due to asthma, or c) the need for additional doses of oral steroids [1]. If there was an exacerbation, the patient or the primary care physician added treatment as indicated in the action plan established at study enrollment. Based on the information recorded in the cooperation records and the Japanese asthma guideline criteria [1], the patient’s control status was classified into one of three categories: patients who experienced one exacerbation or more in 3 years were classified as insufficiently-controlled group (IC). When asthma symptoms occurred less than once a month, or a short acting beta2 agonist (SABA) less than once a month, and when FEV_1_ remains at least 80 % of the predicted value or the person's best value, and there were no nighttime symptoms, or restrictions in daily life activities, patients were categorized as well controlled (WC) [1]. Among well-controlled patients, those who remained asymptomatic for 3 years, required no additional medication, and maintained a %FEV_1_ > 80 % were classified as clinical remission under treatment (CR) [4] (Fig. 1).

During the each yearly collaborative follow-up, FeNO, FOT indices, and spirometry were all assessed in that order. All physiological examinations were performed by clinical technologists.

The FeNO was measured using the NIOX-VERO FeNO analyzer (Chest Co., Tokyo, Japan) according to the instruction manual. With the device in place, the patient was asked to exhale at 50 ml/s after maximum inspiration. The value at which the patient could exhale well was taken as the measured value [12].

The FOT measurements were performed using the MostGraph-01 (Chest Co., Tokyo, Japan) [6]. The device provides continuous respiratory system resistance (Rrs) and impedance (Xrs) measurements in the frequency range of 5–35 Hz. The patients performed rest ventilation while compressing their cheeks to prevent attenuation due to vibration, and measurements were started at a stable stage. The mean value of 5 rest breaths was calculated automatically, and the mean value of inspiration and expiration was used as the measured value. The calculated resistances at 5 Hz and 20 Hz were expressed as R_5_ (cmH_2_O/L/S) and R_20_ (cmH_2_O/L/S), respectively. The impedance at 5 Hz was expressed as X_5_ (cmH_2_O/L/S), the resonant frequency as Fres (Hz), and the integral of reactance up to the resonant frequency as AX (cmH_2_O/L/S * Hz). The mean value of the whole respiratory cycle (inspiration and expiration) was evaluated.

Spirometry was performed using the CHESTAC 8800 (Chest Co., Tokyo, Japan) according to the guidelines for respiratory function tests [13]. The measured FEV_1_ was defined as the FEV_1_ at which maximal expiratory flow was achieved from maximal inspiratory flow and expiratory flow was satisfactory according to the flow volume curve. The FEV_1_ relative to the predicted value was expressed as %FEV_1_.

Statistical analysis was performed using JMP Pro ver. 14 (SAS Institute). The difference between the baseline value and the 3-year follow-up value was evaluated as delta (Δ). Each index was expressed as a frequency or mean and standard deviation, or a median and interquartile range. When the difference between groups was normally distributed, the student t test was performed. Otherwise, the Wilcoxon test was performed. For the association of the frequency of each index, the χ2 test was performed. Spearman’s rank correlation test was performed for the correlation between indices, and the rank correlation coefficient (*ρ*) and *P* value were calculated. In all calculations, *P < 0.05* was regarded as statistically significant. Multiple regression analysis was used to investigate the relationships among ΔFEV_1_ and other parameters in the CR group and the standard partial regression coefficient (*β*) was calculated.

This study complied with the Declaration of Helsinki and was approved by the Ethics Committee of St. Marianna University School of Medicine (No. 4104). This study was a survey of normal clinical examinations. Therefore, the contents of the research were posted at our hospital and on the St. Marianna University website homepage; the research methods were freely available to interested study participants. The Ethics Committee of St. Marianna University School of Medicine waived the requirement for written informed consent owing to the retrospective nature of the study; participants were also given the option to opt-out.

## Results

The baseline demographic characteristics of the 99 participants are shown in Table 1 and Fig. 1. All patients met the criteria for well controlled asthma at baseline. Of all the study participants,17 had at least one exacerbation in 3 years and were assigned to the insufficiently-controlled group (IC). A total of 82 patients met the criteria to categorized well controlled. Of the group, 54 patients met the criteria to be included in the clinical remission under treatment group (CR), and 28 patients did not meet the CR group criteria (WC group) (Fig. 1). Participants met the inclusion criteria for the WC group, if their symptoms were triggered less than once per month and their SABA use was 1-6 times per year. No patients experienced nocturnal symptoms during 3-year study periods. In the IC group (*n*=17), 5 patients required oral steroids, 1 required hospitalization, 3 required unscheduled medical visit due to asthma, 2 needed a step up of their treatment, and 6 needed drug adjustments. All asthma exacerbations in the IC group were mild, and their frequency did not exceed one per year. In the CR group (*n*=54), 4 patients had their treatment stepped down.

The changes in FeNO, FEV_1_, and FOT indices over time are shown in Table 2(a). A significant decrease in FeNO was noted in the 2nd and 3rd years. The FEV_1_ decreased significantly over time, whereas the %FEV_1_ significantly increased. The predicted FEV_1_ decreased significantly. No substantial changes were noted in R_5_ and R_20_. The increase in X_5_ and decreases in Fres and AX were all significant.

**Table 2:**
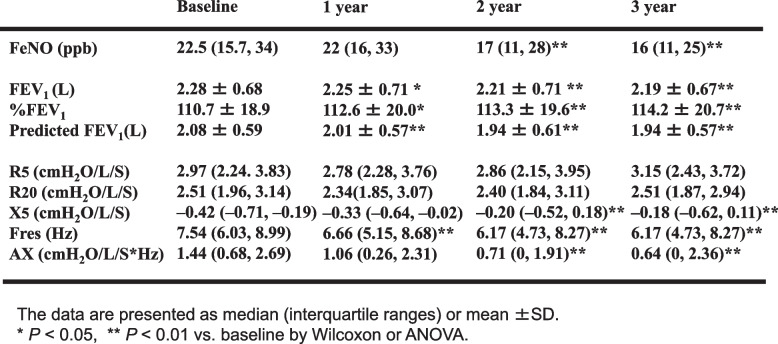
FeNO, FEV_1_, and FOT parameters at baseline, 1 year, 2 year and 3 years

**Fig. 2:**
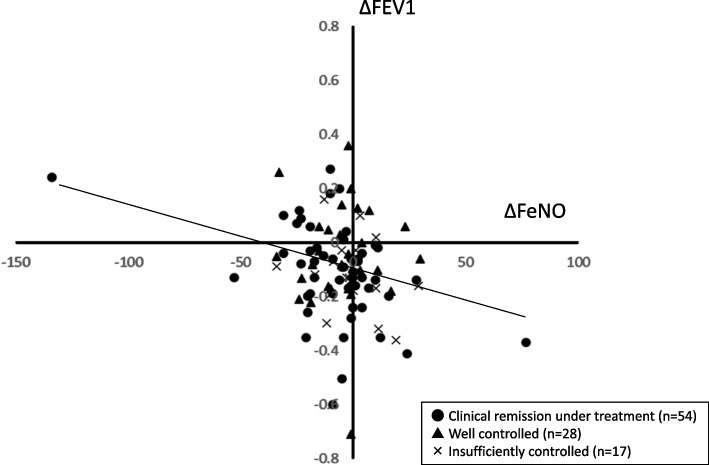
The relationship between ΔFEV_1_ and ΔFeNO. ●: clinical remission under treatment group, ▲: well controlled group, and X: insufficiently controlled group

To observe asthma management over the 3-year study period, we investigated the relationships among indices. Using the difference between the baseline and 3-year measures as delta (Δ), we found a significant negative correlation between Δ FeNO and Δ FEV_1_ (Table 3, Fig. 2), whereas no significant relationship was found between Δ FeNO and changes in the FOT indices. A significant correlation was also found between Δ FEV_1_ and changes in all FOT indices except Δ X_5_. The correlation coefficient was highest for Δ Fres (Table 3).

**Table 3:**
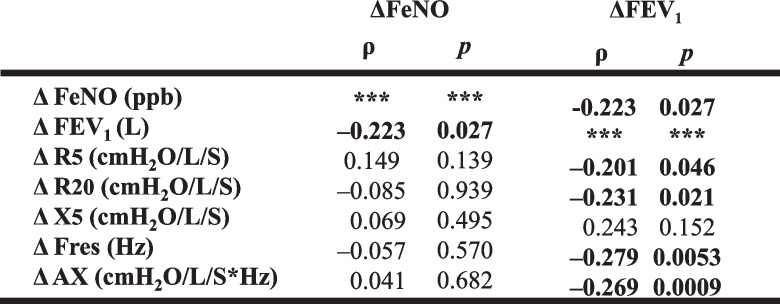
The relationship of change in FeNO, FEV_1_, and FOT parameters over 3 years

At started above, we assigned patients to one of three groups—a) clinical remission under treatment (CR), b) well controlled but not in clinical remission (WC), and c) insufficiently-controlled (IC)—based on the presence of exacerbation symptoms during the 3 years. None of the physiological indices differed significantly among the three groups at baseline (Table 4).

**Table 4:**
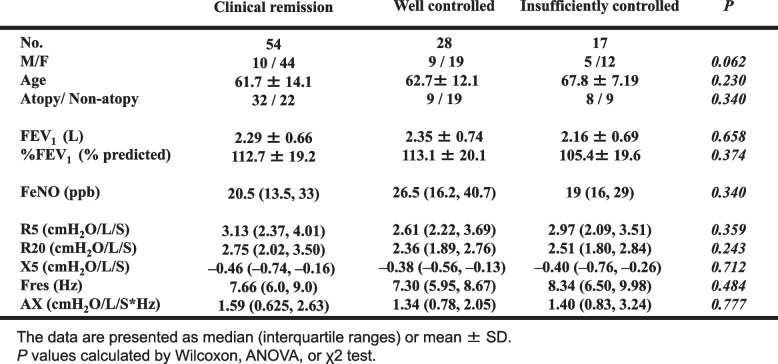
Comparison in FeNO, FEV_1_, and FOT parameters of in the clinical remission group, well controlled group, or insufficiently controlled group at baseline

**Fig. 3:**
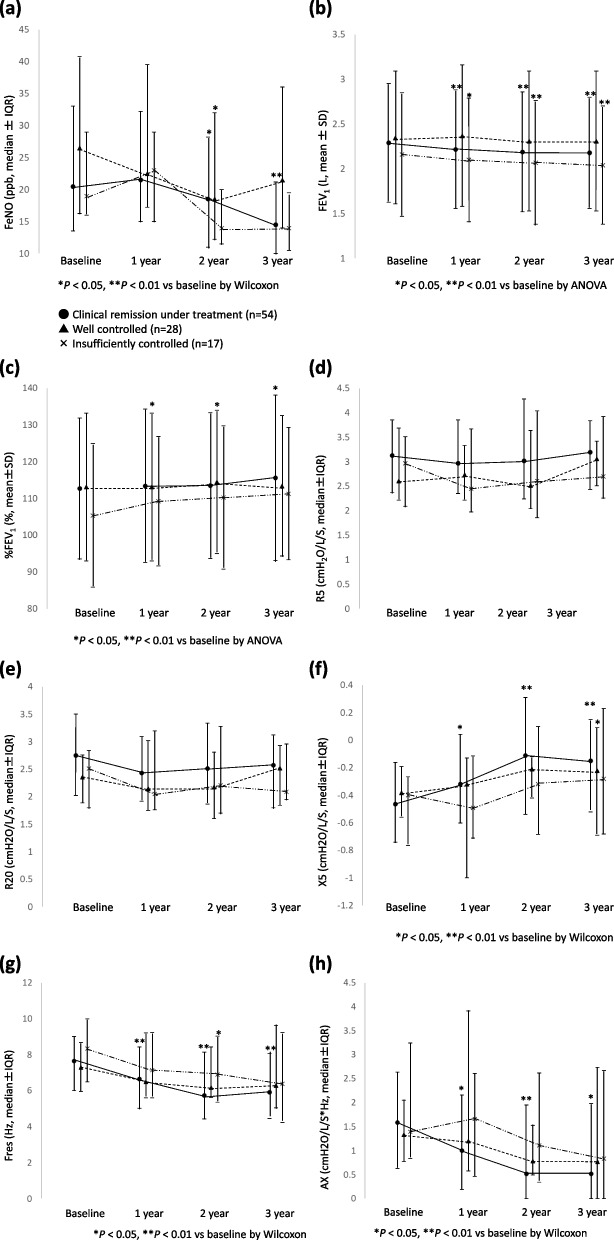
The annual changes in FeNO (a), FEV_1_ (b), %FEV_1_ (c), R_5_ (d), R_20_ (e), X_5_ (f), Fres (g), and AX (h) over 3 years. *P < 0.05 vs baseline, **P < 0.01 vs baseline by ANOVA or Wilcoxon test. ●: clinical remission under treatment group, ▲: well controlled group, and X: insufficiently controlled group

A comparison of the secular changes in each index at annual intervals is shown in Fig. 3. The CR group showed a significant decrease in FeNO in the 2nd and 3rd years, and the WC group showed a significant decrease in FeNO in the 2^nd^ year (Figure3a). The CR group also showed a significant decrease in FEV_1_, whereas no change was observed in the WC group or increased in the IC group (1^st^, 2^nd^, and 3^rd^ year) (Fig. 3b). Conversely, the WC group in years 1 and 2 and the CR group in year 3 showed significant increases in %FEV_1_, whereas no change was seen in the IC group (Fig 3c). In addition, no statistically significant changes in R_5_ and R_20_ were observed in any of the groups (Fig. 3d, e). The CR group in the 1^st^, 2^nd^, 3^rd^ years and WC group in the 3^rd^ year each showed a significant increase in X_5_ (Fig. 3f). Moreover, the CR group in the 1^st^, 2^nd^, 3^rd^ years and IC group in the 2^nd^ year showed significant decreases in Fres (Fig. 3g). The CR group in the 1^st^, 2^nd^, 3^rd^ years also showed significant decreases AX in all 3 years (Fig. 3h). Evaluation of each index demonstrated no significant difference among the 3 groups at baseline or in the 1st, 2nd, and 3rd years.

Table 5 shows the difference in the change in each index among the CR, WC, and IC groups during the 3-year study period. No significant differences in the change in each index were observed despite the presence of exacerbation in the IC group

**Table 5:**
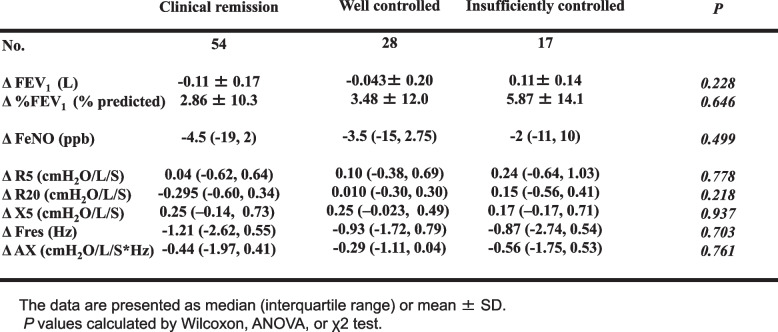
Comparison of change in FeNO, FEV_1_, and FOT parameters in the clinical remission group, well controlled group, or insufficiently controlled group over 3 years

The relationships between the changes in the FOT indices in CR, WC, and IC groups are shown in Table 6. Whereas no significant correlations were found in the WC and IC groups, there were significant relationships between Δ FeNO and Δ FEV_1_, and Δ FEV_1_ and each FOT index change in the CR group. Multiple regression analysis revealed a significant correlation between ΔFEV_1_ and ΔFeNO in the CR group, independently ΔAX , age and sex (Table 7).

**Table 6:**
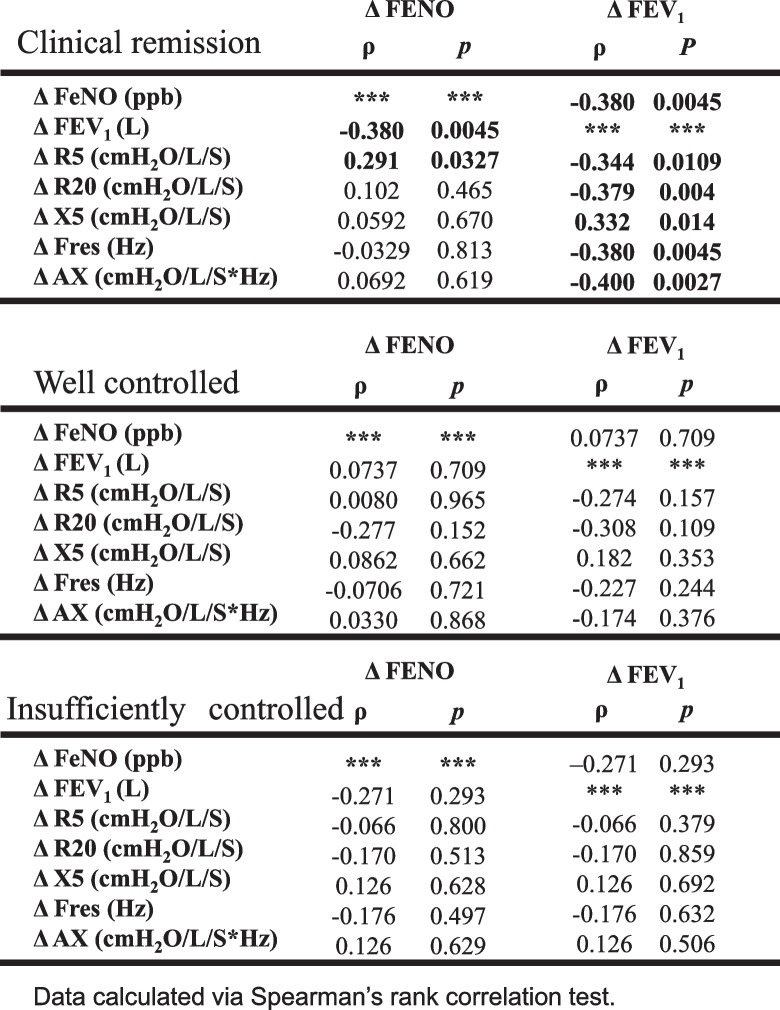
The relationships between the changes in indices in the clinical remission group, well controlled group, or insufficiently controlled group

**Table 7:**
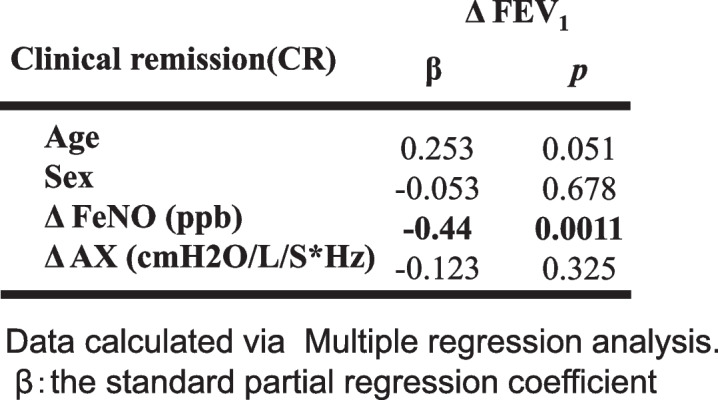
The results of multiple regression analysis in the clinical remission under treatment group

## Discussion

In this study, we examined the changes in FeNO, FEV_1_, and FOT indices over 3 years in 99 patients with asthma managed via a hospital-clinic collaboration system. In our system, all patients with asthma who are well controlled at baseline receive regular examinations by primary care physicians and are managed in our clinics collaboratively with the hospital. Over the 3-year study period, 54 patients maintained CR, whereas 17 patients (17.1%) had exacerbations. This finding underscores the importance of regular reevaluation for patients with controlled asthma.

In the patients we studied, the levels of FeNO tended to decrease over 3 years (Table 3). A previous study [9] reported that FeNO reflected airway inflammation and continuously high levels of FeNO were associated with greater decreases in FEV_1_ and progressive airway remodeling. In our previous study [7], we observed that high FeNO levels also reflected bronchial reversibility in patients with asthma controlled with medication. Interestingly, in the current study, despite a decrease in FEV_1_, the %FEV_1_ tended to increase. Since predicted FEV_1_ decreases with age, the %FEV_1,_ seems to more accurately reflect the patient’s actual condition. The improved airway inflammation and well-maintained respiratory function we observed in our study patients lend additional support to this finding.

Regarding each FOT index, we found no significant trends in R_5_ and R_20_ among the 3 groups; rather, X_5_, Fres, and AX significantly improved in all groups. Previous reports have shown FEV_1_ to be highly correlated with Fres in asthma [14], while X_5_, Fres, and AX correlate highly with airway reversibility [7, 15]. Correlating %FEV_1_ with X_5_, Fres, and AX can be useful for following patients with asthma, and the change in FEV_1_ is related to the change in R_5_ [16]. The difference in X_5_ between inspiration and expiration helps to differentiate asthma from COPD [17]. Therefore, monitoring levels of X_5_, Fres, and AX in patients with asthma seems valid. The differences we observed in R_5_ and R_20_ from those previously reported, may be explained by the effects of age, sex, and body mass index (BMI) [18]. Therefore, the results may be inconsistent and depend on the patient’s characteristics.

The significant correlation between the decrease in FeNO and the increase in FEV_1_ over the 3-year study period (Table 3, Fig. 2) suggests that the control of airway inflammation was reflected in the improved FEV_1_. Moreover, the significant correlations we observed between the FOT indices and Δ FEV_1_, but not Δ FeNO, support previous reports which found no relationship between FeNO and any FOT index [6, 7, 14]. Thus, each index probably reflects a different aspect of asthma.

We found no significant differences among the three groups at baseline (Table 4) and in the change in parameters over 3 years (Table 5, Fig. 3). In addition, there was a significant inverse correlation between Δ FeNO and Δ FEV_1_, and correlations between Δ FEV_1_ and changes in FOT parameters in the CR group, whereas no significant tendencies were observed in the WC and IC groups (Table 6). Our results illustrate that improvement in airway inflammation is associated with improved respiratory function in patients who’ve achieved clinical remission, but the relationship between airway inflammation and respiratory function is less clear in patients whose asthma is not fully controlled. Our findings from multiple regression analysis in the CR group, ΔFEV_1_ correlated independently with ΔFeNO supports prior reports that bronchoconstriction improves if inflammation is suppressed throughout the course of the disease (Table 7). A recent study [3] showed that although correlations between FeNO, FEV_1_, and clinical symptoms have been reported in patients with asthma, the relationships may only persist in patients whose disease is fully controlled.

Evidences also suggests that FeNO and FEV_1_ [19], and FeNO and FOT parameters [20] can predict airway hyperresponsiveness to some extent. Furthermore, modifying treatment based on symptoms, respiratory function, and FeNO helps to prevent future exacerbations in patients with mild-to-moderate asthma [3]. Therefore, we propose evaluating these parameters in combination and keeping them favorable to avoid future exacerbations. Among the FOT indices, Fres and AX seem to reflect the change of FEV_1_ well, especially, in patients with well-controlled asthma managed by primary care physicians as in this study. Therefore, Fres and AX can serve as management indicators. Our previous study showed that Fres and X_5_ levels correlated with bronchial reversibility in patients with low FeNO [7]. A particular advantage of FOT is that it requires minimal patient effort; additional studies are needed to determine its usefulness as an asthma management tool.

The limitations of this study include the following: a) because it was conducted in a single center, the characteristics unique to the western medical care area of Yokohama City are strongly evident and cannot be directly applied to other medical care areas; b) because there are no similar reports, it is not possible to make comparisons to other areas or to perform propensity score matching; c) the study was not designed to determine the effect of the presence or absence of the hospital-clinic collaboration system; d) because treatment is adjusted according to clinical needs during the course of the disease, only the CR group can be said to reflect the natural course of asthma control; and e) because only patients with relatively good compliance can remain in the collaboration system, the status of those who discontinued the program due to complications is unknown; f) Almost patients were well controlled, making it difficult to understand the usefulness of FeNO, spirometry, or FOT. These limitations require additional research.

In conclusion, our study illustrates the need to reevaluate and regularly following patients with well-controlled asthma who are managed by primary care physicians in our hospital-clinic collaboration system to prevent exacerbations and preserve their pulmonary function. In addition, we suggest that FeNO, %FEV_1_, and Fres are useful indices to follow in the ongoing care of patients with asthma.

### Supplementary Information

**Supplementary Material 1.**

## Data Availability

All data generated or analyzed during this study are included in this published article and its supplementary information files.

## Abbreviations

FeNO: fractional exhaled nitric oxide
FEV_1_: forced expiratory volume in 1 second
FOT: forced oscillation technique
R5: the respiratory sistances at 5 Hz
R20: the respiratory sistances at 20 Hz
X5: the impedance at 5 Hz
Fres: the resonant frequency as Fres (Hz)
Ax: the integral of reactance up to the resonant frequency
